# Classification of multiple sclerosis clinical profiles using machine learning and grey matter connectome

**DOI:** 10.3389/frobt.2022.926255

**Published:** 2022-10-13

**Authors:** Berardino Barile, Pooya Ashtari, Claudio Stamile, Aldo Marzullo, Frederik Maes, Françoise Durand-Dubief, Sabine Van Huffel, Dominique Sappey-Marinier

**Affiliations:** ^1^ CREATIS (UMR 5220 CNRS & U1294 INSERM), Université Claude Bernard Lyon1, INSA-Lyon, Université de Lyon, Lyon, France; ^2^ Department of Electrical Engineering, KU Leuven, Leuven, Belgium; ^3^ BIP SpA, Milan, Italy; ^4^ Department of Mathematics and Computer Science, University of Calabria, Rende, Italy; ^5^ Hôpital Neurologique, Service de Neurologie, Hospices Civils de Lyon, Bron, France; ^6^ CERMEP–Imagerie du Vivant, Université de Lyon, Lyon, France

**Keywords:** multiple sclerosis, brain connectivity, grey matter, machine learning, artificial intelligence–AI

## Abstract

**Purpose:** The main goal of this study is to investigate the discrimination power of Grey Matter (GM) thickness connectome data between Multiple Sclerosis (MS) clinical profiles using statistical and Machine Learning (ML) methods.

**Materials and Methods:** A dataset composed of 90 MS patients acquired at the MS clinic of Lyon Neurological Hospital was used for the analysis. Four MS profiles were considered, corresponding to Clinical Isolated Syndrome (CIS), Relapsing-Remitting MS (RRMS), Secondary Progressive MS (SPMS), and Primary Progressive MS (PPMS). Each patient was classified in one of these profiles by our neurologist and underwent longitudinal MRI examinations including T1-weighted image acquisition at each examination, from which the GM tissue was segmented and the cortical GM thickness measured. Following the GM parcellation using two different atlases (FSAverage and Glasser 2016), the morphological connectome was built and six global metrics (Betweenness Centrality (BC), Assortativity (*r*), Transitivity (T), Efficiency (*E*_*g*_), Modularity (Q) and Density (D)) were extracted. Based on their connectivity metrics, MS profiles were first statistically compared and second, classified using four different learning machines (Logistic Regression, Random Forest, Support Vector Machine and AdaBoost), combined in a higher level ensemble model by majority voting. Finally, the impact of the GM spatial resolution on the MS clinical profiles classification was analyzed.

**Results:** Using binary comparisons between the four MS clinical profiles, statistical differences and classification performances higher than 0.7 were observed. Good performances were obtained when comparing the two early clinical forms, RRMS and PPMS (F1 score of 0.86), and the two neurodegenerative profiles, PPMS and SPMS (F1 score of 0.72). When comparing the two atlases, slightly better performances were obtained with the Glasser 2016 atlas, especially between RRMS with PPMS (F1 score of 0.83), compared to the FSAverage atlas (F1 score of 0.69). Also, the thresholding value for graph binarization was investigated suggesting more informative graph properties in the percentile range between 0.6 and 0.8.

**Conclusion:** An automated pipeline was proposed for the classification of MS clinical profiles using six global graph metrics extracted from the GM morphological connectome of MS patients. This work demonstrated that GM morphological connectivity data could provide good classification performances by combining four simple ML models, without the cost of long and complex MR techniques, such as MR diffusion, and/or deep learning architectures.

## 1 Introduction

An important issue in neuroscience is the characterization of human brain structure and function, and their alteration in brain diseases. Today, the neurologist’s challenge is to define disease phenotypes based on their underpinning mechanisms and to predict the disease evolution. Multiple Sclerosis (MS), which is the most common chronic immune-mediated disabling neurological disease affecting the central nervous system (Steinman, 1996; Goodin, 2014), is primary classified according to clinical symptoms. In about 85% of cases, disease onset is characterized by a first acute episode, called Clinically Isolated Syndrome (CIS), it evolves to a Relapsing-Remitting course (RRMS) followed by a Secondary-Progressive course (SPMS) The remaining 15% of MS starts directly from a Primary-Progressive course (PPMS) (Ghasemi et al., 2016; Lublin et al., 2014; McDonald et al., 2001). However, the clinical course of the disease and the risk for developing permanent disability are very different from one patient to another (Scalfari et al., 2010; Lynch et al., 2005). MS is characterized by pathological processes including inflammation and demyelination, leading to lesions, predominantly in White Matter (WM) tissue, that can be detected by conventional T2-weighted MRI (Compston and Coles, 2008). Nevertheless, lesions are also present in Grey Matter (GM) as initially demonstrated by histochemical studies (Geurts and Barkhof, 2008; Calabrese et al., 2013), and more recently by high field MRI (Bruschi et al., 2020; Tallantyre et al., 2009). These findings, as well as the measurements of GM atrophy (Filippi, 2015; Durand-Dubief et al., 2012), have confirmed the neurodegenerative hypothesis in MS (Ghasemi et al., 2016; Steenwijk et al., 2016; Preziosa et al., 2017). Kuceyeski et al., 2018 showed that GM atrophy is associated with the highest prediction accuracy of the patient’s future processing speed in MS. More recently, cortical thickness has been recognized as an early marker of neurodegeneration in MS (Cruz-Gomez, 2021).

In the last decade, Artificial Intelligence (AI) approaches have been increasingly applied within the medical field, hoping to increase diagnostic performance and improve treatment. Specifically, Machine Learning (ML) is a data-driven approach which covers a very broad set of methods. Indeed, ML aims to extract possibly complex relations among available data and generate predictions for an event. A wide range of ML applications have been proposed in the literature. A systematic review of the applications of ML methods in autoimmune diseases is proposed by Stafford et al. (2020). In a recent review paper, Segato et al. (2020) highlighted that AI methods nowadays are among the most widely used analytical tools, while classical ML approaches, such as support vector machines and random forest, are still widely used. Notwithstanding, in the field of MS, ML approaches have often focused on automatic examination of MRI images to classify disease at the time of onset or to predict evolution of clinically isolated forms (Kaka et al., 2020; Afzal et al., 2020). Such an example is proposed by Jackson et al. (2020), where a Genetic Model of MS Severity (GeM-MSS) was used for the evaluation of MS disability progression considering a cohort of 426 MS patients and obtaining a Root Mean Squared Error (RMSE) of 0.46 and a correlation with MS-DSS score of 0.21. Kolčava et al. (2020) proposed a classification task for predicting a second clinical event in 64 MS patients with a CIS and based on a multicenter MRI dataset. Logistic regression and Cox proportional hazards regression models were used, obtaining a sensitivity and specificity score of 84% and 63% respectively. In Table 1 a tabular review of the most relevant studies of ML application in the field of MS is provided. For a thorough review of ML applications in MS we also refer to Vázquez-Marrufo et al. (2021) and Garavand et al. (2022). Notwithstanding, DL models are becoming increasingly more important in biomedical diagnostics. For example, for the task of Epileptic Seizures Detection, multiple DL methods have been implemented and summarized in Shoeibi et al. (2021a). Also, in Shoeibi et al. (2021c), various intelligent DL-based methods for automated Schizophrenia diagnosis were described using electroencephalography signals (EEG). Interestingly, in the context of MS, multiple DL techniques are proposed for the task of disease detection, segmentation and classification using MRI data and summarized in Shoeibi et al. (2021b).

**TABLE 1:** Overview of the most relevant studies in the field of MS analysis from the literature using MRI data.

Author	Dataset	Method	Application	Performance criteria
Seccia et al. (2020)	1515 MS	SVM; RF; ADB; KNN; RNN	Conversion from RR to SP	Spec = 86.2%;
				Sens = 84.1%;
				Acc = 86.2%
Barile et al. (2022)	90 MS	Boosting Ensemble	Disability estimation	RMSE = 0.92
Rubinov and Sporns (2010)	90 MS	CNN, GraphNN	MS Classification	RMSE = 0.09
Rosa et al. (2018)	105 MS	Cascade of Two 3D	Segmentation	DSC=60; VD=40
		Patch-Wise CNNs		
Brichetto et al. (2020)	810 MS	LR, SVM, KNN	Conversion from RR to SP	Acc = 82.6%
Zhao et al. (2020)	724 MS	LR, SVM, RF	EDSS prediction after 5 years	Spec = 69%;
				Sens = 79%;
				Acc = 71%;
				AUC = 78%
Pinto et al. (2020)	187 MS	KNN, DT, LR, SVM	Conversion from RR to SP	Spec = 77%;
				Sens = 76%;
				AUC = 86%
Afzal et al. (2021)	19 MS	Two 2D-CNN	Segmentation; Lesion Detection	DSC=67%
				Sen=48%
				Pre = 90%
Fleischer et al. (2020)	12 MS; 12 HC	CBN; TPDC	Causal Effects between brain areas	-
Shrwan and Gupta (2021)	38 MS	2D-CNN	MS Classification	Acc = 99.55%;
				Pre = 99.15%
Ye et al. (2020)	38 MS	DNN	MS Classification	Acc = 93.4;
				Sen = 99.1
Barile et al. (2021a)	48 MS	GAN	MS Classification	F1 = 81%
Barile et al. (2021b)	70 MS	NTF	MS Classification	F1 = 76%
Yperman et al. (2020)	642 MS	LR; RF	Disability Prediction	Acc = 75%;
				AUC = 75%
Pruenza et al. (2019)	48,186 MS	RF	MS progression	Acc = 82%

SVM, Support Vector Machines; RF, Random Forest; LR, Logistic Regression; ADB, Adaboost; KNN, K-Nearest Neighbour; CNN, Convolutional Neural Network; RNN, Recurrent Neural Network; DNN, Deep Neural Network; GAN, Generative Adversarial Network; GraphNN, Graph Neural Network; NTF, Non-Negative Tensor Factorization; HC, Healthy Control; MS, Multiple Sclerosis; RR, Relapsing-Remitting MS; SP, Secondary-Progressive MS; CBN, Causal Bayesian Network; TPDC, Time-resolved Partial Directed Coherence; RMSE, Root Mean Squared Error.

Graph theory represents a new and powerful approach for characterizing brain networks by providing both global and local metrics (Rubinov and Sporns, 2010; Guo et al., 2017), using either functional MRI or diffusion tensor imaging (DTI). Recently, Tozlu et al. (2021) demonstrated that dynamic functional and structural connectome metrics outperformed results obtained from conventional MRI clinical data when discriminating MS patients by impairment level. Previously, Kocevar et al. (2016) demonstrated the interest of DTI structural connectivity for the classification of MS clinical profiles using ML methods. Marzullo et al. (2019) improved the classification performance by using a CNN model. Schiavi et al. (2022) used structural disconnection for the classification of multiple sclerosis patients considering 55 MS patients and 24 healthy controls. Five different classifiers were used reaching an accuracy in the range between 64.5% and 91.1%. However, fMRI and DTI data, used for connectivity modeling, require long acquisition time and the use of complex processing techniques, strongly limiting their applicability in clinical practice. Nevertheless, brain connectivity can also be obtained from conventional MRI by measuring different morphological metrics of the GM on T1-weighted images (Raamana and Strother. 2018). In such graphs, nodes represent GM areas obtained from the GM tissue parcellation, while edges represent a degree of (dis-)similarity between nodes using features like GM thickness (MacDonald et al., 2000). Indeed, these morphological graphs follow a small-world topology at a macroscale level, characterized by a high degree of local clustering and short path-lengths linking individual network nodes (He et al., 2007). Such an approach has been recently used in Alzheimer’s Disease (AD), showing that a GM network measures predicted hippocampal atrophy rates within individuals with preclinical AD, in contrast to other AD biomarkers (Dicks et al., 2020). In MS, Muthuraman et al. (2016) analyzed morphological GM thickness networks in order to classify CIS and RRMS patients using Support Vector Machines model, obtaining good level of accuracy.

The main goal of this study is to investigate whether cortical thickness atrophy, in patients affected by MS, represents a discriminative biomarker for MS profiling. Our hypothesis is based on previous studies where the GM thickness morphometric feature was demonstrated to be one of the most important biomarkers characterizing MS patients (Durand-Dubief et al., 2012). Also, focusing on network changes and not on local structural properties may represent an important step forward for a better discrimination of the MS clinical profiles. In order to test such an hypothesis, an ensemble of four ML models was used for the classification of MS patients based on the morphological GM connectivity using the GM thickness feature. The graph characterization was based on six global graph metrics, describing the topological behavior of the connectome. Two different spatial resolutions were considered for GM regional parcellation, which allows to test the initial hypothesis in two different settings (i.e., high and low spatial resolution). Also, with the aim of investigating whether the choice of the parcellation atlas has an impact on our initial hypothesis, the results obtained from the statistical and ML analysis were compared. To our knowledge, this is the first attempt to characterize brain networks of MS patients based on their GM atrophy and to perform statistical analysis and automatic classification of MS clinical profiles using two parcellation approaches. An ensemble of 4 ML models was considered in order to improve the binary classification task, while taking into account the thresholding impact on the topological architecture of the GM network.

## 2 Materials and methods

### 2.1 Study population and MRI acquisition

In this study, 90 MS patients were examined at different time points, every 6 months during the first 3 years and then every year for the following 4 years. Patients were recruited at the MS clinic of Lyon Neurological Hospital and underwent a MR examination at the CERMEP MRI department, on a 1.5T S Sonata system (Siemens Medical Solution, Erlangen, Germany) using an 8-channel head-coil. The MR protocol included the acquisition of a sagittal 3D-T1 sequence (1 × 1 × 1 *mm* ^3^, *TE*/*TR* = 4/2000 *ms*). A total of six-hundred-fifty-two scans were obtained, corresponding to 12 CIS, 30 RRMS, 28 SPMS, 20 PPMS. To better clarify the dataset used in this study, Table 2 reports the summary information for all the MS patients. This study was approved by the local Ethics Committee (CPP Sud-Est IV) and the French national agency for medicine and health products safety (ANSM). Written informed consent was obtained from all patients prior to study initiation.

**TABLE 2:** Summary information of the dataset partition into MS clinical profiles (CIS, RR, SP, PP) and healthy subjects. Average values for Age (at first scan) and Disease Duration (DD) with standard deviation in parentheses is reported. Median values of EDSS is provided along with range of variation in parentheses. Percentage of female patients is reported.

	N Patients	N Scans	Age	DD	Sex (%)	EDSS
CIS	12	64	32.4 (6.4)	3.0 (1.9)	47	1 (0–3)
PP	20	140	40.4 (6.3)	7.5 (2.9)	66	4 (2.5–6.5)
RR	30	233	33.6 (7.1)	8.3 (4.9)	82	2.5 (0–4.5)
SP	28	215	40.8 (4.8)	14.9 (6.0)	40	5 (3–7)

### 2.2 GM connectivity generation

**FIGURE 1:**
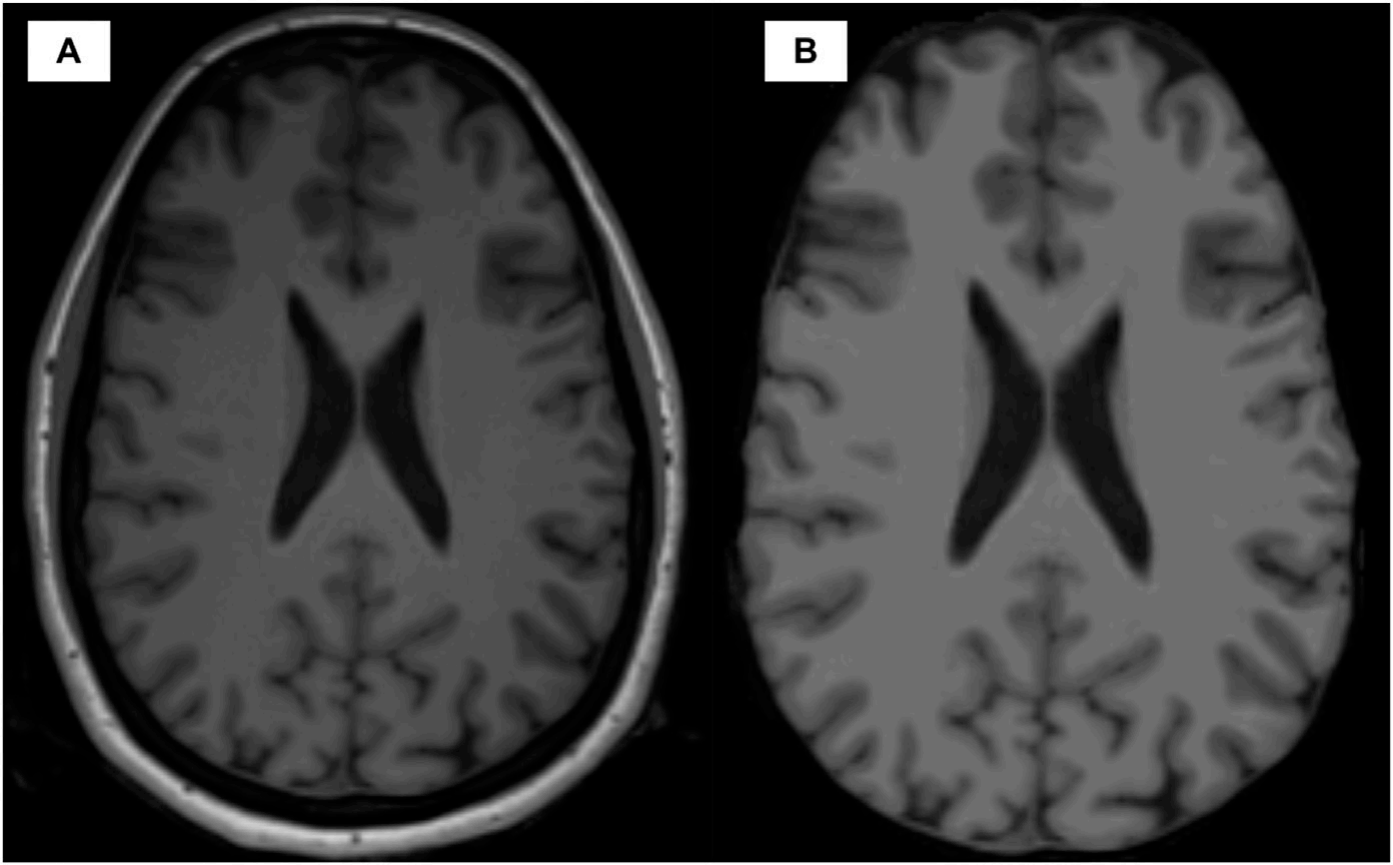
Visual comparison between the anatomical T1w image modality before **(A)** and after **(B)** preprocessing.

**FIGURE 2:**
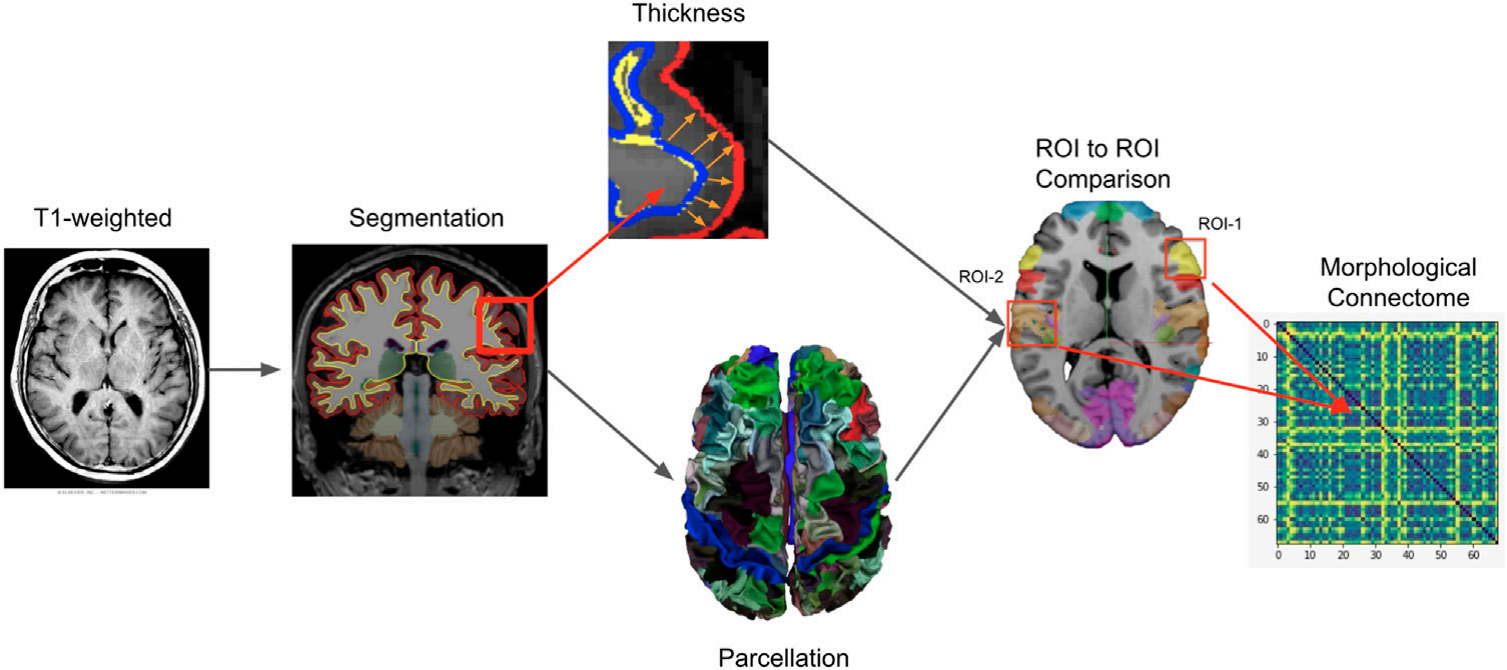
Intuitive representation of the pipeline implemented for morphological connectome data generation. Starting from the magnetic resonance T1-weighted image of a patient, the GM tissue was first segmented and then parcelled in different anatomical brain regions (ROIs) based on a specific atlas used as template (FSAverage or Glasser 2016). Additionally, from the segmented tissue, the thickness morphometric statistic was calculated for each GM region. A ROI to ROI binary comparison was performed for all possible combinations of GM regions (one to one comparison). The Manhattan distance formulation was used as dissimilarity metric. The results were organized in a squared symmetric adjacency matrix (i.e. connectome) where each entry represent the (dis-)similarity between two ROIs. From the connectome, six global metrics were calculated for the analysis of graph characterization.

Starting from 3D T1-weighted images, several preprocessing steps were applied to the anatomical image of each patient and based on the Freesurfer v6.0.0 image analysis suite (Reutera et al., 2012). A detailed description of all the preprocessing steps can be found in Hanganu et al. (2015). In particular, the skull was removed from the brain image and motion correction as well as registration and intensity normalization were performed in order to reduce noise and make all the MRI scans comparable, avoiding pixel intensity artifacts that may distort the final analysis. Additionally, the MRI image was resampled into a 3D coordinate system called the Talairach space. It allows to map the location of brain structures such that MRI scans with different size and overall shape of the brain are mapped using comparable 3D coordinates. To better visualize the impact of the preprocessing, Figure 1 is shown to illustrate the effect for a random subject sampled from our cohort of MS patients. Finally, segmentation of the anatomical tissue, including cortical and sub-cortical GM segmentation was performed. Each voxel was classified into one of four classes, such as WM, cortical GM, sub-cortical GM and cerebro-spinal fluid (CSF). The cortical thickness morphometric feature was calculated for each GM pixel and clustered in brain regions based on the used parcellation atlas. The exact pipeline used to build the morphological connectome was described in Raamana and Strother (2017). The reliability of the pipeline was validated using histological (Rosas et al., 2002) and manual measurements (Rosas et al., 2002) demonstrating high performances across different scanner and field of strengths (Reutera et al., 2012). Additionally, previous studies have performed similar analysis demonstrating the robustness of the implemented pipeline (Muthuraman et al., 2016; Kuperberg et al., 2003). The GM parcellation task was performed using two different atlases, the FSAverage (Rosenke et al., 2017; Fischl et al., 1999) and the Glasser 2016 (Glasser et al., 2016), providing 68 and 360 brain regions (nodes), respectively. The GM morphological connectome of each patient was obtained comparing the morphometric features (i.e., GM thickness) of each brain region, using the Manhattan distance formulation (Craw, 2017), and expressed by a full squared symmetric matrix $A\in R^{q\times q}$ , where *q* represents the number of nodes (brain regions). The corresponding graph representation of this adjacency matrix can be defined as *G* = (*V*, *E*, *ω*) where *V* represents the set of nodes defining brain regions (|*V*| = *q*), *E* represents the set of edges between these regions (|*E*| = *m*) and *ω* defines the strength of association (Raamana and Strother, 2017; Raamana and Strother, 2018). An intuitive representation of the pipeline implemented for morphological connectome data generation is shown in Figure 2.

### 2.3 Graph binarization

In order to perform the brain morphological connectivity analysis, graphs were binarized to remove the weakest connections generated by the morphometric GM thickness comparison. Henceforth, a percentile threshold 0 < *τ* < 1 was imposed and a corresponding binarized unweighted and undirected graph $\tilde{G}=\left(\tilde{V},\tilde{E}\right)$ was obtained, where $\left|\tilde{V}|=q=|V\right|$ and $|\tilde{E}|=\tilde{m}\leq m$ . It is worth noticing that the topology and density of the network are affected by the imposed threshold (Bullmore and Sporns, 2009; Simpson et al., 2013). In this work, a proportional thresholding approach was applied to determine the binarization value. Mathematically, this is equivalent to impose a function $\Phi :G\to \tilde{G}$ . Let’s define with *vec*(*A*_*i*_) the vectorization of the matrix A for scan *i* of a specific patient and with $vec{\left(A_{:}\right)}_{++}$ the concatenation of *vec*(*A*_*i*_)*∀i* ∈ *K* where *K* represents the set of all patients’ scans. The total percentile distribution over all vectorized concatenation of all MRI scans can be defined as $d_{vec{\left(A_{:}\right)}_{++}}$ . Based on this formulation, a proportional thresholding strategy can be applied. Basically, each absolute value Φ, corresponding to the associated percentile *τ* form distribution $d_{vec{\left(A_{:}\right)}_{++}}$ in the range 5%–95% at steps of 5%, was considered as a valid binarization score. Formally speaking, for each percentile threshold *τ*, a function Φ can be defined as:

$$\Phi =\left\{\begin{array}{cc} 0,\; & \text{if}\;\omega <\phi \\ 1,\; & \text{otherwise} \end{array}\right.$$

For the analysis, we followed the same approach proposed by Kocevar et al. (2016). The Coefficient of Variation (CV) was used as measure of variability.

### 2.4 Graph metrics estimation

In this study, six global graph features were calculated based on the binarized connectome $\tilde{G}$ of each patient’s scan. Graph Density (D) (Barnes, 1969) is defined as the ratio between the numbers of connections in the graph over the number of possible connections. It represents the easiest graph metric and it intuitively conveys information about the “density of connections” between nodes. Maximum value of D will be obtained when every node is connected with any other node in the network. Assortativity (*r*) (Thedchanamoorthy et al., 2014) represents the correlation coefficient between the degrees of two nodes at the extremities of an edge. It quantifies the tendency of a network to have individual nodes connected with other similar nodes (Newman, 2002) and thus it can be intuitively quantified as a Pearson correlation. Transitivity (T) (Bouwer, 1972) is the ratio between the number of triangles, defined as triplets of nodes interconnected, and the number of all possible triplets in the graph. Intuitively, T represents the overall probability for the network to have adjacent nodes interconnected, thus revealing the existence of tightly connected communities also known as clusters. Interestingly, complex networks with small-world properties often have high transitivity. Global Efficiency (*E*_*g*_) (Ek et al., 2015) is the mean of the inverse of the shortest path distance in the graph between each pair of nodes. Intuitively, such a metric represents the efficiency to which information is propagated through the network. In fact, it measures the average propensity to reach a node *j* from a node *i* in as less number of steps (involved nodes) as possible. Interestingly, the change of efficiency caused by failures or attacks can be used to assess the robustness or resilience of networks (Zhou and Wang ,2018). Average Betweenness Centrality (BC) (Jannoud, 2014) is defined as the total number of shortest paths from node *i* to node *j* that pass through a specific node *v* of interest over the total number of possible shortest paths, averaged with respect to all the nodes in the network. In other words, the BC metric measures the extent to which a vertex lies on paths between other vertices. Intuitively, vertices with high BC values may have considerable influence within the network since they “control” (i.e. influence) the overall information traveling between nodes. Interestingly, nodes with high BC are also the ones which mostly disrupt communication between vertices when removed from the network, since they represent a “bridge” between two communicating nodes and their disruption increases the number of steps required for the information to travel, ultimately reducing the efficiency of the network. Modularity (Q) (Lancichinetti and Fortunato, 2009) describes the capability of a network to be separated into modules. Such modules are also known as communities and they are characterized by the appearance of densely connected groups of vertices, with only sparser connections between groups. Thus, the detection of modules inside the human brain may be an interesting feature for studying interdependence between Regions of Interests (ROIs) (Newman, 2006). For an in-depth description and application of these metrics we refer to Stamile et al. (2021).

## 3 Statistical and machine learning analysis

### 3.1 Statistical analysis

MS clinical profiles were compared based on their global graph metrics using Generalized Linear Model (GLM) with binomial family and logistic link (Agresti, 2021). Stata16 statistical programming language (StataCorp, 2021) was used along with the command *xtmelogit* for GLM model fitting. The longitudinal aspect of the dataset was taken into account by considering both mixed and random effects. The evaluation of statistical differences between MS clinical profiles, for each global graph metric, was performed controlling for age and gender as confounding factors. The longitudinal aspect of the dataset was taken into account by the random effect, which model the time component, while the remaining regressors were included as fixed effects. The tests were computed with a level of significance of 5%.

### 3.2 Classification of MS clinical profiles

**FIGURE 3:**
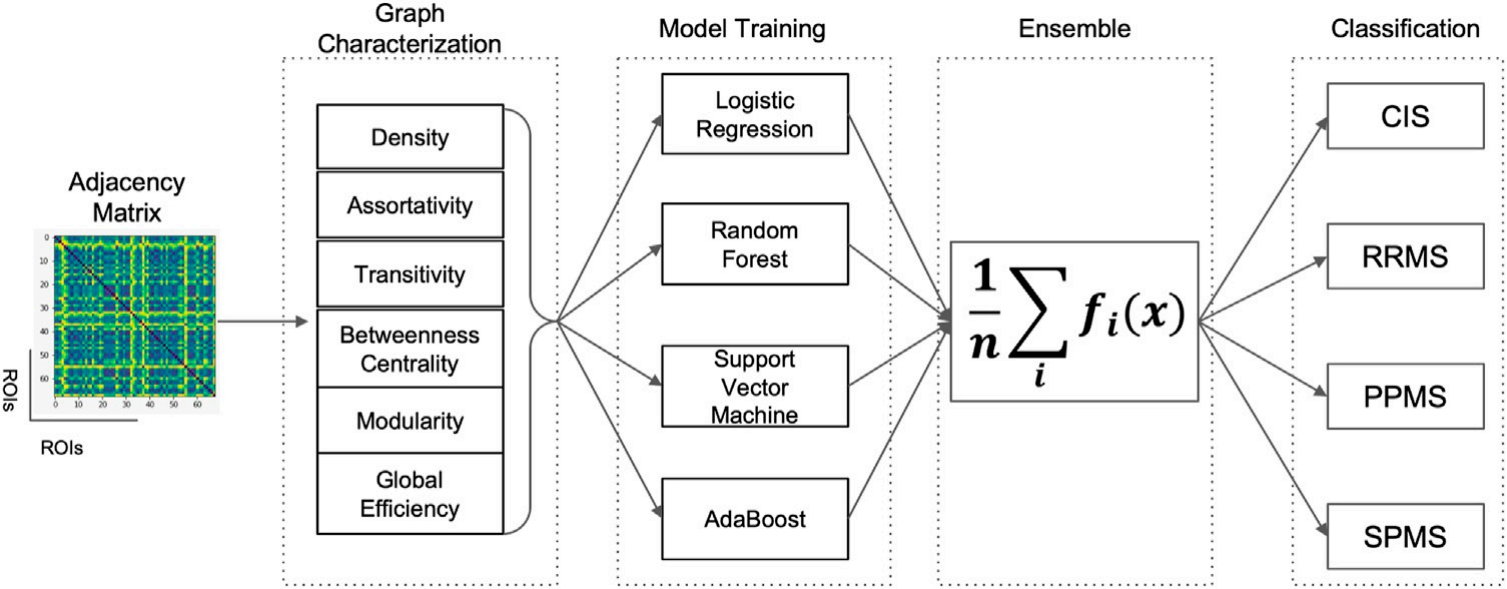
Schematic representation of the Machine Learning pipeline implemented for the classification of MS clinical profiles. Starting from the adjacency matrix representation of the brain connectome for a patient, six global graph metrics (Betweenness Centrality (BC), Assortativity (*r*), Transitivity (T), Efficiency (*E*_*g*_), Modularity (Q) and Density (D) were calculated and used as input to the four Machine Learning models (Logistic Regression, Random Forest, Support Vector Machine and AdaBoost) independently trained. The four models were then combined in a final ensemble model by majority voting and then used for binary classification.

Four different ML models were considered in this study for the classification task, namely Logistic Regression (LR) (Cox, 1958), Random Forest (RF) (Breiman, 2001), Support Vector Machine (SVM) (Cortes and Vapnik, 1995) and Adaptive Boosting (AdaBoost) (Schapire, 2013) models. The predictive analysis was performed using Python 3.6 programming language along with *scikit-learn* package v0.24.2, while the *networkx* package v2.2 was used for global graph metrics calculation. The 4 ML models were chosen as they are widely used, easy to train and tune with a minimal number of hyperparameters to optimize during cross validation (Witten et al., 2011) and provide built-in feature importance for the prediction task, with the exception of SVM in case a non-linear kernel is used (i.e., Radial Basis Function Kernel). Additionally, simple models are usually preferred with small datasets to avoid overfitting the training set (Ghojogh and Crowley, 2019). Moreover, the different underlying assumptions, used for each classifier, may provide interesting insights for the classification performance. A combination of many different predictors can often improve predictions (Sollich and Krogh, 1995), and in statistics this idea has been investigated extensively (Granger, 1989). In order to boost the performances and exploit the individual properties of each classifier, we combined them into a higher level ensemble model through majority voting. To better clarify the ML pipeline, Figure 3 was proposed. Predictive performances were compared considering the non-parametric Wilcoxon matched-pairs signed-rank test.

### 3.3 Statistical metrics

In this work, five statistical parameters were used for the predictive analysis, such as accuracy, precision, recall, and F1. Such metrics are based on the analysis of true positive (TP), true negative (TN), false positive (FP), and false negative (FN) instances classified during the test phase (Gorunescu, 2011). Formally speaking, accuracy is defines as the ratio between the number of correct assessments and the number of all assessments $\left(\frac{TP+TN}{TP+TN+FP+FN}\right)$ , precision defines the fraction of retrieved instances that are correctly classified $\left(\frac{TP}{TP+FP}\right)$ and recall identifies the portion of positive instances that are correctly identified $\left(\frac{TP}{TP+FN}\right)$ , also known as sensitivity. Additionally, F1 score is obtained combining precision and recall and is defined as $2*\frac{Precision*Recall}{Precision+Recall}$ . Finally, the Area Under the Receiver Operating Characteristics (ROC-AUC) curve score was also calculated for completeness. Our validation method consists of a stratified 10 fold cross-validation strategy. More precisely, at each iteration, one fold was left out as test set. From the remaining instances, 80% were used as training set and 20% as validation set. The training set was used for parameter tuning and the model was validated on the validation set to avoid overfitting. The entire procedure was repeated 10 times and the results averaged. It is important to notice that in order to avoid data leakage, if a specific patient was assigned to the test set, all its corresponding longitudinal scans were also selected.

### 3.4 Model optimization

In order to optimize the models, a grid search strategy was employed. In particular, the following hyperparameters were optimized. For the LR model a regularization parameter in the interval between 1 and 100 was used, while for the RF model, the number of trees were optimized in the range between 10 and 500 and the max depth of each tree between 1 and 10. For the SVM model, a radial basis kernel (RBF) was used due to its good performance, as demonstrated in previous studies (Kocevar et al., 2016), along with a regularization parameter and kernel coefficient in the range between 1 and 100. Finally, for the AdaBoost model, the number of trees was tuned in the range between 10 and 500, with a learning rate between 0.1 and 3. Additionally, the thresholding value (*τ*), used for graph binarization, as explained in Section 2.3, was also considered as an additional hyperparameter along the entire percentile range. In order to avoid data leakage, the search for the optimal threshold was only performed on the training set. The final results reported in Section 4 refers to the performance obtained on the hold-out test sets.

## 4 Results

### 4.1 Descriptive statistical analysis of GM connectivity

**FIGURE 4:**
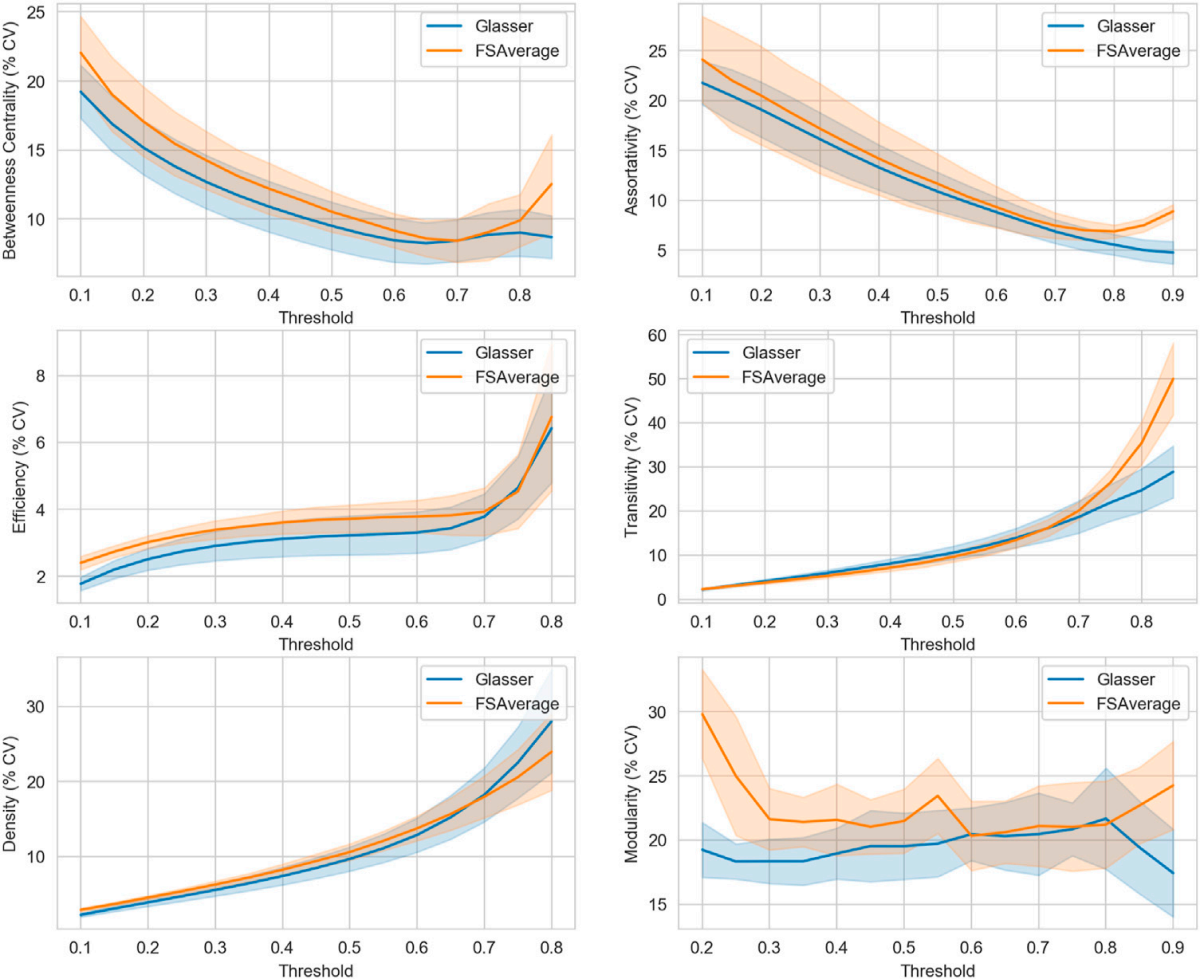
Variability study over the entire percentile threshold range, using two different atlases [FSAverage (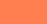) and Glasser 2016 (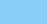)]. Each block defines the Coefficient of Variation (% CV), expressed in percentage values (y-axis), at each specific percentile threshold in the range [0–1] (x-axis) for a defined global graph feature. For visual purposes, the upper and lower threshold values were cut off at a convenient value in order to avoid image flattening.

Morphological GM connectivity was characterized using six global graph metrics and two different atlases for parcellation. By measuring the degree of variability (i.e. CV coefficient) along different percentile thresholding values, for each graph metrics (Figure 4), a similar behavior was observed with both atlases. This first result showed that the spatial resolution of parcellation did not have a strong impact on the variability of graph topology. Second, we observed that the smallest CV score was obtained for a *τ* value comprised between 0.7 and 0.8 for the graph metrics BC and *r*. These metrics play an important role in describing the information flow of the network and the stability of centralized hubs. Also, the variability of the modularity (Q) metric was coherent and stable between the two atlases only in the range of 0.6–0.8. This result is important as this metric is crucial for maintaining the small-world property characterizing the human brain (Sporns and Betzel, 2016). For the remaining three global metrics, namely *E*_*g*_, T and D, an opposite trend was observed. For *E*_*g*_ and T metrics an exponential increase in the CV coefficient was observed for a value of *τ* greater than 0.7. Overall, these results suggest that the information flow between nodes far apart inside the network, and possibly pertaining to different modular clusters, start to be compromised for higher thresholding values, due to the reduction of intra-cluster connections and lower number of triangular motifs (reduced local transitivity). Notwithstanding, a stable value of CV in the range of *τ* between 0.4 and 0.7 can be noticed for the *E*_*g*_ metric. Finally, the D metric increased steadily until a value of *τ* equal to 0.7 from which an increased rate was observed, particularly for the Glasser 2016 atlas which provides high spatial resolution parcellation. Therefore, we concluded that good topological characteristics could be observed for a value of *τ* in the range between 0.6 and 0.8. For this reason, the statistical analysis in Section 4.2 was performed considering a binary thresholding value of 0.7 as a reasonable compromise since it represents the central value of the suggested range.

### 4.2 Descriptive statistical analysis of MS clinical profiles

**FIGURE 5:**
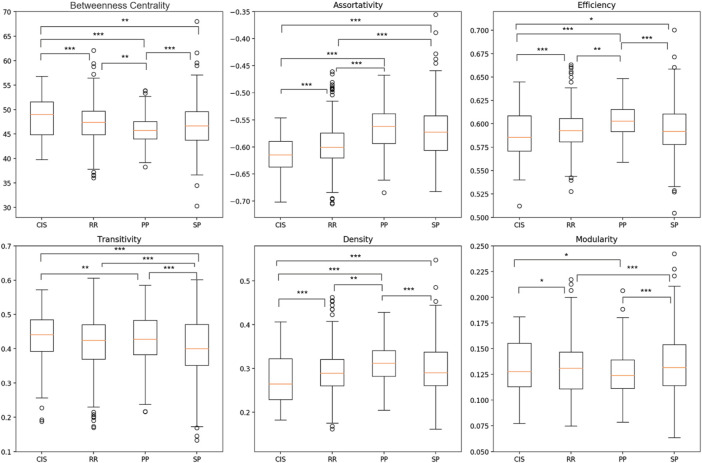
Boxplot comparison between four MS clinical profiles over six global graph metrics using FSAverage atlas. Differences between clinical profiles were determined employing a generalized mixed effect model with age and sex as controlling factors (*p < 0.05; **p < 0.01; ***p < 0.001).

**FIGURE 6:**
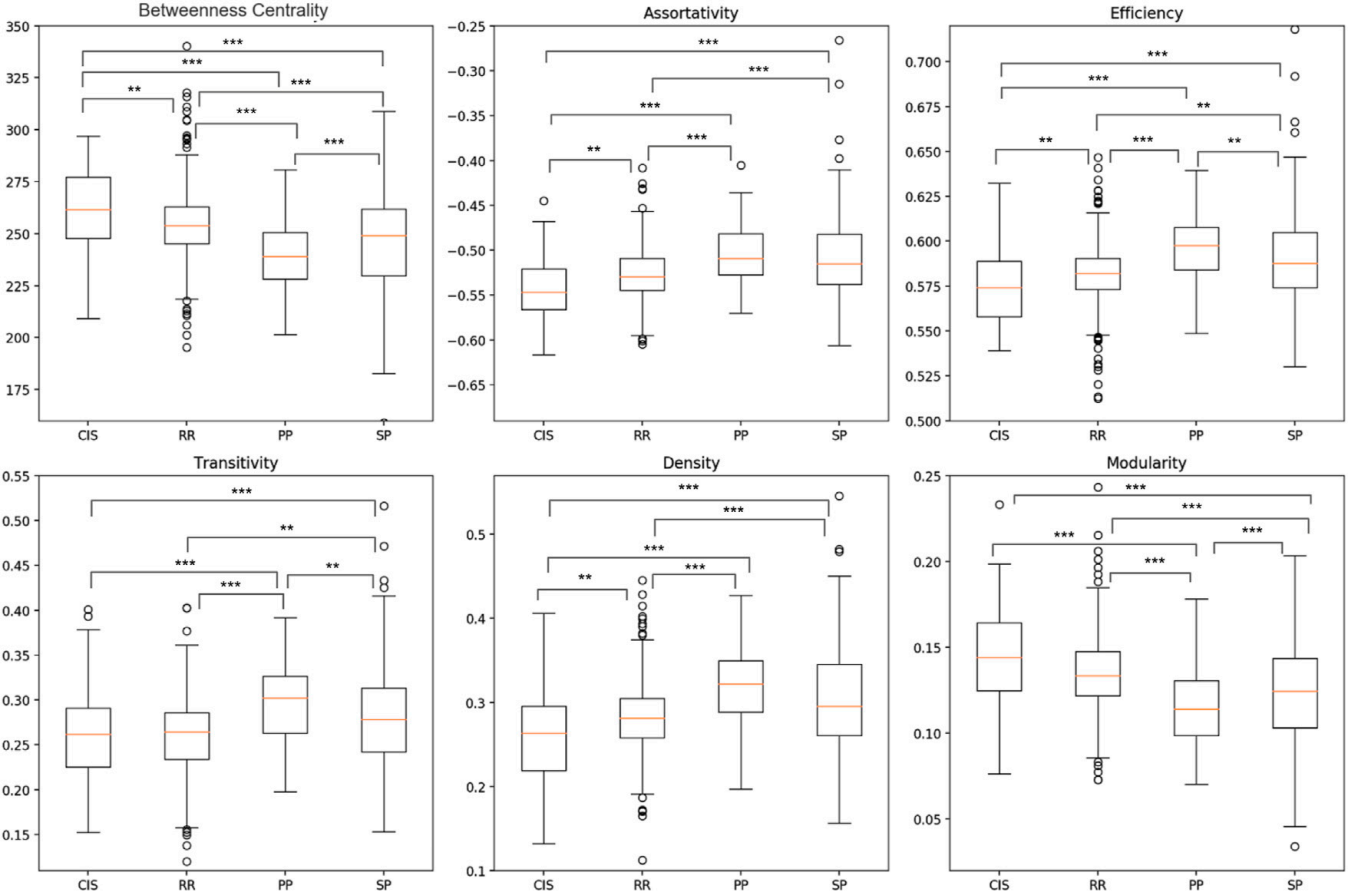
Boxplot comparison between four MS clinical profiles over six global graph metrics using Glasser 2016 atlas. Differences between clinical profiles were determined employing a generalized mixed effect model with age and sex as controlling factors (*p < 0.05; **p < 0.01; ***p < 0.001)

Statistical analysis of the four MS clinical profiles was performed by characterizing graphs with six global metrics calculated using two atlases, FSAverage and Glasser 2016. As shown in Figure 5 and Figure 6, significant differences were detected between the MS clinical profiles for most of the graph metrics, especially considering the higher spatial resolution (Glasser 2016) atlas. This is evident when comparing the Transitivity (T) and Modularity (Q) metrics, for which the Glasser 2016 atlas reported significant differences for almost all binary comparisons, with the exception of the CIS and RRMS groups. This result showed that a more refined parcellation may be helpful in the discrimination of clinical profiles. Considering the Betweenness Centrality (BC) metric, a significant reduction was observed comparing patients in the beginning stages of the disease, such as CIS and RRMS, with progressive MS courses (PPMS and SPMS). Conversely, for *r*, *E*_*g*_ and D, reduced mean values were observed for CIS and RRMS patients compared to progressive courses (*p* < 0.01). When comparing CIS with progressive courses, significant differences (*p* < 0.05) were detected for almost all metrics, with the exception of Q using the FSAverage atlas. However, only small differences were observed between CIS and RRMS patients. Additionally, significant differences (*p* < 0.05) were observed between PPMS and SPMS groups, especially when the Glasser 2016 atlas was used. From these results, it is possible to conclude that GM thickness was more significantly altered in progressive and degenerative courses, compared to earlier MS stages. The brain networks of PPMS and SPMS patients exhibited a reduced number of centralized nodes, leading to randomized distribution of the information transfer.

### 4.3 Classification of MS Clinical profiles

**FIGURE 7:**
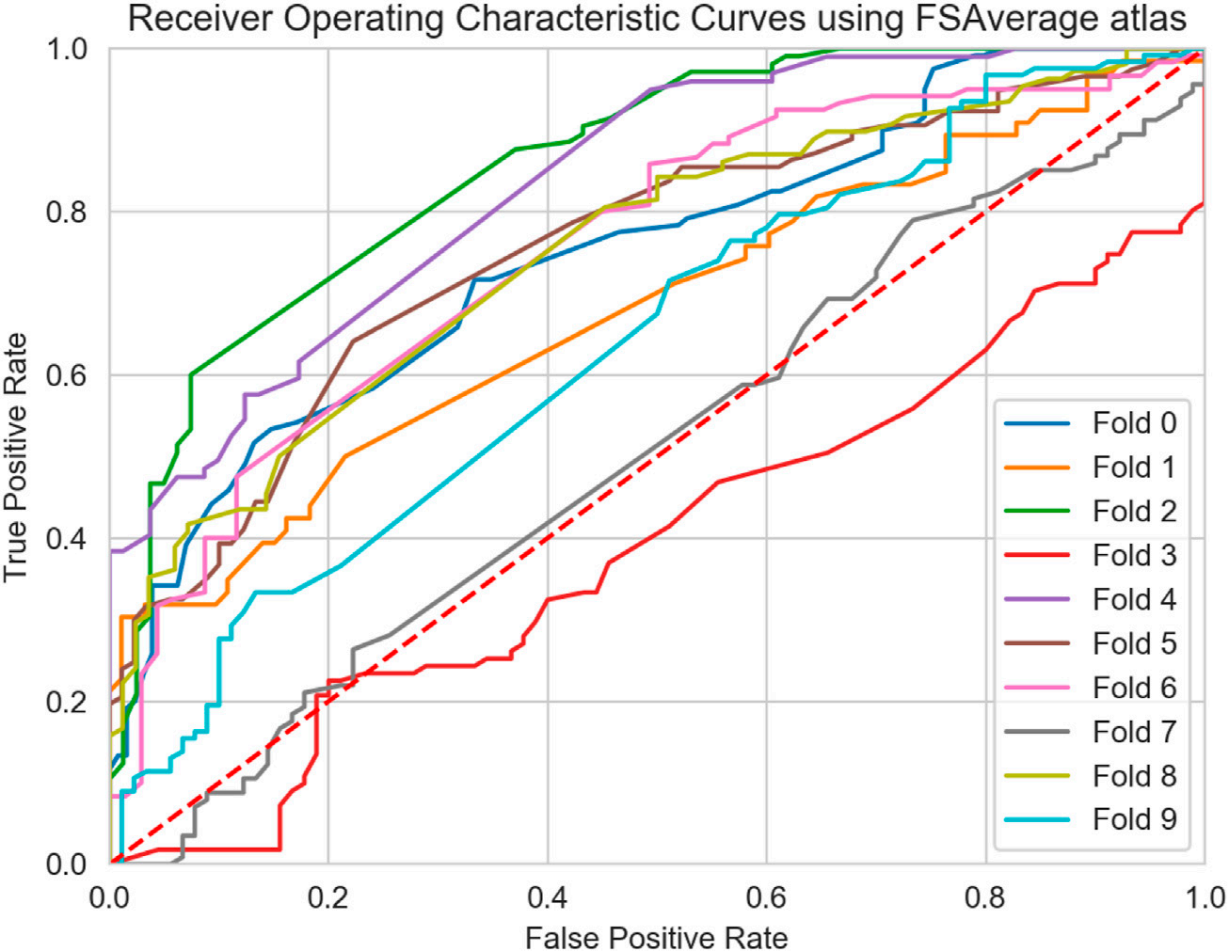
Receiver Operating Characteristic Curves (ROC) curve of the ensemble model for all 10-folds of the cross validation using the FSAverage atlas.

**FIGURE 8:**
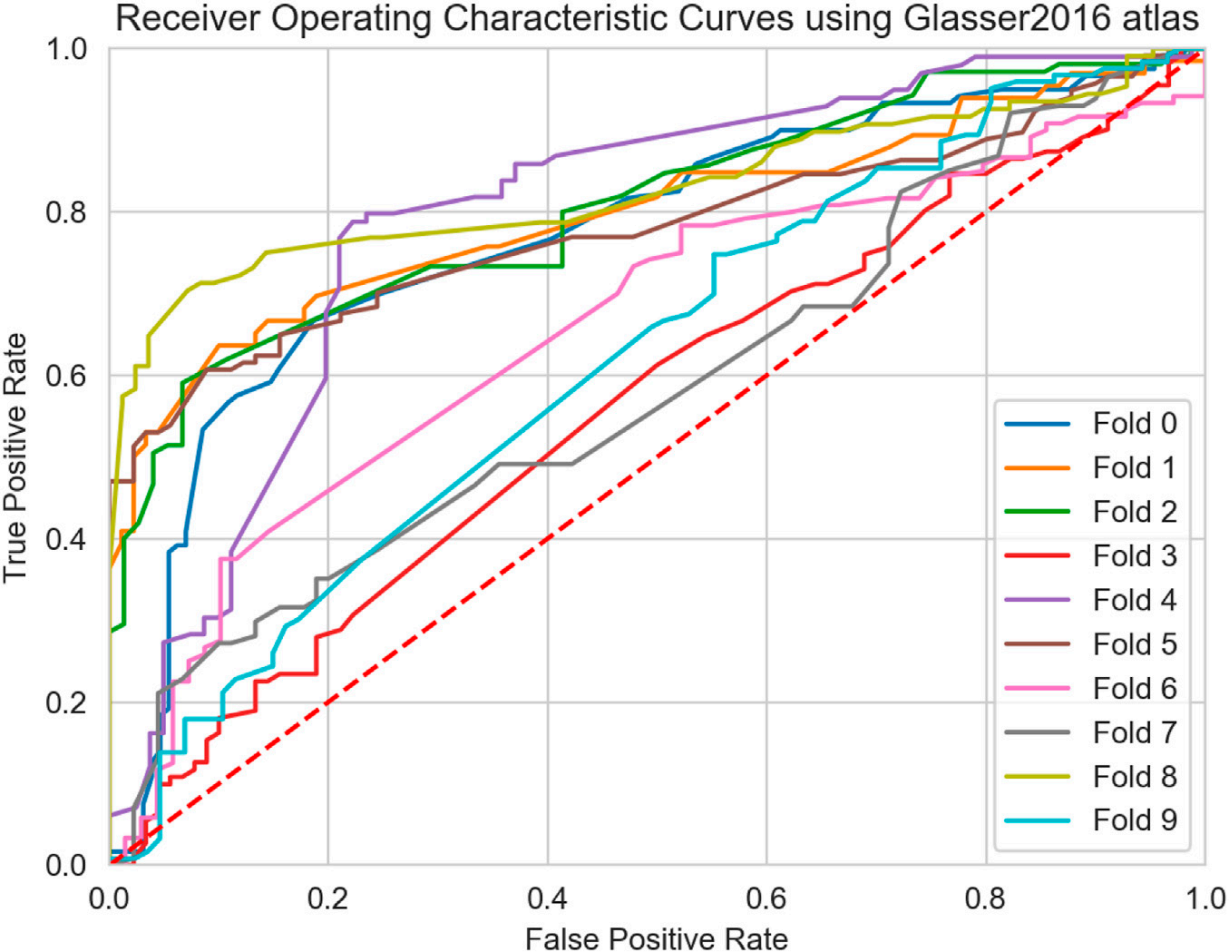
Receiver Operating Characteristic Curves (ROC) curve of the ensemble model for all 10-folds of the cross validation using the Glasser 2016 atlas.

From the descriptive statistical analysis, significant differences were detected, suggesting good degree of separation between MS clinical profiles. Thus, in order to evaluate the predictive performance of the six global graph metrics, the 4 ML models were trained separately and subsequently combined in an ensemble model, by late integration using majority voting, as described in Section 4.2. The classification task was performed using the two atlases separately and the results compared in Tables 3, 4. Overall, comparable results were observed between the two atlases. Moreover, high classification performance scores (*F*1 > 0.7 for both atlases) were obtained comparing patients in the primary stage of the disease (CIS), with progressive courses (PPMS and SPMS). When considering the AUC score, a similar classification performance (0.75) between CIS and PPMS was observed with the FSAverage atlas while lower score (0.67) was obtained using the Glasser 2016 atlas, when comparing CIS and SPMS groups. These findings suggested that a more detailed GM parcellation may lead to less informative connectomes and a reduced classification performance. Interestingly, when comparing CIS with RRMS patients, no significant difference (*p* > 0.05) in performance was observed between the two atlases (FSAverage; 0.66 and Glasser 2016; 0.58). This result showed the limit of GM connectome to discriminate patients in the early stages of the disease, as CIS and RRMS profiles are mainly subject to inflammation and less degeneration leading to GM atrophy especially when a large (less refined) parcellation strategy was considered. Notwithstanding, the high level of imbalance between the two groups as well as the low number of CIS patients considered in this study may play a substantial role. Conversely, when comparing RRMS patients with PPMS, first, significant differences were detected comparing the FSAverage and Glasser 2016 atlases (*p* < 0.05), with an AUC score of 0.70 and 0.84 respectively, conform with the statistical analysis in Section 4.2. Surprisingly, when comparing RRMS with SPMS patients, poor classification performances were detected, especially when the Glasser 2016 atlas was considered (AUC score of 0.62). This result is probably due to the high variability between the two clinical profiles, as observed from the boxplot analysis in Figure 5 and Figure 6. This result did not significantly differ from the one obtained using the FSAverage (AUC score of 0.67) atlas. For the comparison between progressive courses, a good level of classification (F1 score of 0.72) was obtained using the Glasser 2016 atlas, compared to the less refined FSAverage atlas (F1 score of 0.66). Also, the AUC scores were similar reporting a value of 0.67 and 0.72 for the FSAverage and Glasser 2016 atlas, respectively. Interestingly, the performance, as expressed by the F1 score and AUC scores, obtained from the two parcellation strategies, provided an interesting insight for the use of a more refined parcellation approach, although no differences were detected (*p* > 0.05). As an additional ablation study, in comparison with previous reports (Muthuraman et al., 2016; Kocevar et al., 2016), the classification results obtained using only the SVM model were reported for both atlases in Tables 5, 6. Of particular relevance is the fact that the SVM model alone outperformed the ensemble model when the RRMS group was compared with progressive courses (PPMS: AUC score of 0.79 and SPMS: AUC score of 0.71), using the FSAverage atlas. However, when Glasser 2016 atlas was used, this result was not confirmed when RRMS and PPMS patients were compared (F1 score of 0.58 and AUC score of 0.59). A second experiment was also performed in order to investigate the classification performances obtained by comparing early stages of MS patients (CIS and PPMS) with progressive MS patients (PPMS and SPMS). For this comparison, Figures 7, 8 are proposed, which depict the Receiver Operating Characteristic (ROC) curves for both atlases and for each of the folds in the cross-validation. From these Figures, it can be observed that both atlases display a high variability between the folds. Hence, no firm conclusions can be drawn when comparing the two parcellation strategies. Notwithstanding, the lower performances obtained for the FSAverage atlas are mainly due to two out of 10 folds, namely fold 3 and 7, for which very low classification performance of the ensemble model was obtained. As shown in Tables 7, 8, multiple MS profile comparisons were considered using the FSAverage and Glasser 2016 atlas, respectively. Notwithstanding, good level of classification performances (AUC) were observed considering the CIS and RR group combined, with respectively PPMS (FSAverage: 0.65; Glasser 2016: 0.69) and SPMS (FSAverage: 0.66; Glasser 2016: 0.69) patients. However, no statistical differences were observed between the two atlases. Conversely, when comparing RRMS patients with progressive courses, significant differences (*p* < 0.05) were observed between the two atlases with higher score (F1 and AUC scores of 0.78) obtained with FSAverage. Also, it is interesting to compare this result with the one obtained when considering CIS and RRMS together against progressive MS courses. In this case, an F1 score of 0.65 was obtained for both atlases, suggesting that the inclusion of CIS patients happened to be harmful for the classification performance, probably due to the high variability and the low number of patients in the CIS group.

**TABLE 3:** Mean (st.dev) of the predictive performances of the ensemble model across the ten folds of the cross validation using FSAverage atlas.

Group	F1	Precision	Accuracy	AUC
CIS-RR	0.653 (0.19)	0.737 (0.17)	0.626 (0.21)	0.575 (0.24)
CIS-PP	0.782 (0.19)	0.869 (0.14)	0.789 (0.18)	0.805 (0.16)
CIS-SP	0.753 (0.15)	0.826 (0.13)	0.733 (0.16)	0.749 (0.18)
RR-PP	0.725 (0.14)	0.733 (0.14)	0.743 (0.12)	0.695 (0.13)
RR-SP	0.667 (0.07)	0.699 (0.07)	0.678 (0.06)	0.674 (0.07)
PP-SP	0.663 (0.11)	0.708 (0.13)	0.665 (0.11)	0.673 (0.13)

**TABLE 4:** Mean (st.dev) of the predictive performances of the ensemble model across the ten folds of the cross validation using Glasser 2016 atlas.

Group	F1	Precision	Accuracy	AUC
CIS-RR	0.762 (0.11)	0.811 (0.09)	0.747 (0.12)	0.657 (0.15)
CIS-PP	0.834 (0.12)	0.872 (0.09)	0.819 (0.13)	0.798 (0.14)
CIS-SP	0.723 (0.07)	0.809 (0.07)	0.693 (0.07)	0.672 (0.09)
RR-PP	0.858 (0.09)	0.883 (0.06)	0.868 (0.08)	0.836 (0.11)
RR-SP	0.602 (0.10)	0.637 (0.11)	0.624 (0.08)	0.616 (0.10)
PP-SP	0.719 (0.09)	0.735 (0.08)	0.716 (0.09)	0.724 (0.09)

**TABLE 5:** Ablation study: Mean (st.dev) of the predictive performances of the SVM model across the ten folds of the cross validation using FSAverage atlas.

Group	F1	Precision	Accuracy	AUC
CIS-RR	0.624 (0.15)	0.695 (0.13)	0.603 (0.17)	0.504 (0.20)
CIS-PP	0.771 (0.15)	0.836 (0.14)	0.788 (0.12)	0.758 (0.17)
CIS-SP	0.734 (0.15)	0.799 (0.14)	0.717 (0.16)	0.705 (0.21)
RR-PP	0.793 (0.09)	0.808 (0.09)	0.795 (0.09)	0.787 (0.10)
RR-SP	0.705 (0.07)	0.711 (0.07)	0.706 (0.07)	0.706 (0.07)
PP-SP	0.646 (0.09)	0.702 (0.09)	0.618 (0.09)	0.618 (0.10)

**TABLE 6:** Ablation study: Mean (st.dev) of the predictive performances of the SVM model across the ten folds of the cross validation using Glasser 2016 atlas.

Group	F1	Precision	Accuracy	AUC
CIS-RR	0.752 (0.09)	0.785 (0.07)	0.737 (0.11)	0.603 (0.14)
CIS-PP	0.831 (0.13)	0.872 (0.10)	0.814 (0.15)	0.795 (0.16)
CIS-SP	0.724 (0.06)	0.812 (0.08)	0.693 (0.06)	0.671 (0.12)
RR-PP	0.801 (0.08)	0.805 (0.07)	0.803 (0.07)	0.791 (0.09)
RR-SP	0.582 (0.08)	0.586 (0.09)	0.591 (0.07)	0.585 (0.08)
PP-SP	0.735 (0.08)	0.743 (0.08)	0.733 (0.08)	0.733 (0.09)

**TABLE 7:** Ablation study: Mean (st.dev) of the predictive performances of the ensemble model across the ten folds of the cross validation using FSAverage atlas and multiclass binary comparisons.

Group	F1	Precision	Accuracy	AUC
CIS+RR-PP	0.644 (0.13)	0.672 (0.13)	0.645 (0.12)	0.654 (0.13)
CIS+RR-SP	0.632 (0.12)	0.659 (0.13)	0.633 (0.12)	0.644 (0.12)
CIS+RR-PP+SP	0.649 (0.11)	0.681 (0.12)	0.651 (0.11)	0.663 (0.11)
RR-PP+SP	0.778 (0.09)	0.792 (0.10)	0.778 (0.10)	0.774 (0.10)

**TABLE 8:** Ablation study: Mean (st.dev) of the predictive performances of the ensemble model across the ten folds of the cross validation using Glasser 2016 atlas and multiclass binary comparisons.

Group	F1	Precision	Accuracy	AUC
CIS+RR-PP	0.661 (0.12)	0.745 (0.06)	0.671 (0.11)	0.694 (0.08)
CIS+RR-SP	0.658 (0.12)	0.731 (0.06)	0.668 (0.10)	0.691 (0.08)
CIS+RR-PP+SP	0.648 (0.11)	0.721 (0.06)	0.658 (0.10)	0.681 (0.08)
RR-PP+SP	0.704 (0.09)	0.761 (0.08)	0.704 (0.08)	0.732 (0.08)

## 5 Discussion

GM atrophy is known to occur in MS, even at the earliest stages of the disease (Eshaghi et al., 2018) and may vary between brain regions. Since GM atrophy is probably less affected by inflammation, these GM alterations may provide a more reliable marker of neurodegeneration in MS. Moreover, measuring the cortical thickness may constitute a more sensitive marker than traditional volume-based measures as previously demonstrated (Narayana et al., 2013; Nygaard et al., 2015).

### 5.1 Classification of MS forms

Recalling that our initial hypothesis was that of evaluating the discrimination power of cortical thickness atrophy, graph theory techniques were employed for testing such an hypothesis. Specifically, the power of graph representation was combined with the anatomical GM thickness feature. Six of the most important global graph metrics were used and based on a previous study (Kocevar et al., 2016). Also, an ML analysis was performed using an ensemble of four different ML models. A previous study attempted similar tasks (Muthuraman et al., 2016) by characterizing GM network data considering only CIS and RRMS profiles, thereby reaching good level of accuracy (97%) using an SVM model. The present study extended the comparison to all MS phenotypes and proposed a fully automated pipeline to generate GM connectivity graphs, using only the anatomical images. To our knowledge, this is the first attempt to classify all the clinical MS phenotypes considering both a statistical and ML approach. Our pipeline is particularly relevant for use in clinical practice since our method is only based on classical anatomical T1w images, which represent the most common modality in clinical applications because of its fast and cheap acquisition. From the obtained results, when comparing CIS with progressive courses (PPMS and SPMS), good level of classification performances were obtained, in agreement with our initial hypothesis as well as a previous study (Muthuraman et al., 2016). In fact, CIS represents the first stage of the disease, usually characterized by tissue inflammation, while PPMS and SPMS correspond to the progressive evolution of the disease where severe GM tissue degeneration occurs. Notwithstanding, poor predictive performances were obtained comparing CIS and RRMS clinical profiles, particularly when the FSAverage atlas was used. Thus, our initial hypothesis does not hold for MS patients in the primary inflammatory stages of the disease, meaning that thickness alone was not able to discriminate patients according to these two MS profiles. However, the lack of discrimination between the two early stages of MS may be explained by the reduced number of patients in the CIS group and the high level of imbalance with respect to the RRMS profile, which represents the most common MS group (Zahoor et al., 2017). Additionally, interesting results were obtained comparing multiple global metrics. In particular, patients in the early inflammatory stages of the disease (CIS and RRMS) showed a more assortative brain structure compared with patients in progressive stages (PPMS and SPMS). Such a measure evaluates the behavior of strongly correlated cortical regions, in terms of cortical thickness. From a clinical standpoint, a value closer to zero may be indicative of patterns of cortical degeneration between highly correlated GM regions (i.e. ROIs). In fact, cortical degeneration in specific regions of the GM tissue reduces the thickness properties of such regions compared to those not-affected, reducing the overall assortativity of the network. The exact opposite was found in previous studies in which connectome data was obtained from WM streamline tractography (Kocevar et al., 2016). This result is expected due to the opposite information provided by GM and WM graphs. Indeed, while the former measures the degree of dissimilarity between 2 GM cortical regions (dissociative measure), the latter measures the axonal interconnectivity between two brain regions by counting the number of streamline fibers obtained from tractography (associative measure), thus providing opposite interpretations.

As long as node centrality and modularity measures are concerned, a reduction of such metrics was observed in patients with progressive courses, especially when high resolution parcellation was considered (Glasser 2016). These results may suggest that the small-world property of the human brain was altered. Advances in connectomics and network neuroscience have found that the small-worldness of brain networks is associated with efficient communication (Bullmore and Sporns, 2012). Thus, from a clinical standpoint, such observations suggest that when small-worldness is disrupted, the communication between different GM regions becomes less efficient, in agreement with results already found in the literature (He et al., 2009; Fleischer et al., 2017). Interestingly, once again this result is in agreement with the WM analysis performed by Kocevar et al. (2016) and with our initial hypothesis. It is important to notice that, these aspects were taken into account by the ML model. In fact, for the FSAverage atlas, BC and *r* turned out to be the overall most important metrics, pointing to strong predictive discrimination. Moreover, a higher *E*_*g*_ and D were observed in later stages of MS, consistent with the concept of progressive neural loss and consequently of structural hubs, thereby increasing the randomness of the global network (Rimkus et al., 2018). All these findings are coherent with our initial hypothesis stating that pathological cortical thickness alteration may represent an important biomarker for measuring the degree of degeneration between different MS profiles. Additionally, due to the high number of statistically significant differences between clinical profiles, observed for both atlases (Figure 5 and Figure 6), it is reasonable to question whether single global graph metrics can naively classify MS patients in their respective clinical profiles. However, from the boxplot analysis and from the results reported in Table 9, we can conclude that a trivial binary thresholding cannot discriminate MS profiles and more sophisticated ML models are required.

**TABLE 9:** Mean (st.dev) values of global metrics [Betweenness Centrality (*BC*), Assortativity (*r*), Transitivity (T), Efficiency (*E*_*g*_), Modularity (Q) and Density (*D*)] were calculated on binarized graphs for *τ*=0.7 using FSAverage and Glasser 2016 atlas.

FSAverage Atlas						
	BC	*r*	*E*_*g*_	T	D	Q
CIS	48.83 (3.83)	−0.62 (0.04)	0.58 (0.02)	0.44 (0.08)	0.27 (0.05)	0.13 (0.03)
RR	47.81 (3.94)	−0.60 (0.04)	0.59 (0.02)	0.41 (0.08)	0.29 (0.05)	0.13 (0.03)
PP	44.28 (3.14)	−0.57 (0.05)	0.60 (0.02)	0.42 (0.08)	0.33 (0.05)	0.12 (0.02)
SP	46.20 (4.94)	−0.57 (0.05)	0.59 (0.03)	0.39 (0.09)	0.31 (0.06)	0.13 (0.03)
**Glasser 2016 Atlas**						
CIS	263.76 (23.97)	−0.55 (0.04)	0.57 (0.02)	0.26 (0.06)	0.25 (0.06)	0.15 (0.03)
RR	254.73 (19.19)	−0.53 (0.03)	0.58 (0.02)	0.26 (0.04)	0.28 (0.05)	0.14 (0.02)
PP	239.77 (16.56)	−0.50 (0.03)	0.60 (0.02)	0.30 (0.04)	0.33 (0.05)	0.11 (0.02)
SP	242.86 (25.11)	−0.51 (0.04)	0.59 (0.03)	0.28 (0.06)	0.32 (0.07)	0.13 (0.03)

### 5.2 Atlas comparison

Two different parcellation atlases were considered in this study in order to investigate the impact of the spatial resolution of the GM regions. From the analysis performed in Section 4, only the comparison between RRMS and PPMS resulted in significant differences in performance between the two atlases. The lack of significant differences in all the remaining binary comparisons might be explained by the low number of patients and the high variability associated with the obtained results. Notwithstanding, clear evidence in favor of the high spatial resolution atlas (Glasser 2016) can be noticed with generally higher classification scores in terms of both F1 and AUC scores. Conform with the statistical analysis, the Glasser 2016 atlas showed high degree of variation between MS clinical profiles, most likely due to the larger size of the connectome due to more precise GM parcellation. Notwithstanding, the morphological features extracted from each GM region are subject to larger variability since a reduced number of pixels was considered for creating each link inside the connectome. For this reason, a larger number of patients might be needed in order to confirm our results.

### 5.3 Limitations of the study

This work has also some limitations. First, the reduced number of patients in our dataset, especially for the CIS group, may provide some degree of uncertainty in the generalization results. This has led to an imbalanced dataset when comparing the CIS group with other clinical profiles. In order to tackle the problem, weights were imposed to the cost functions of each ML model in order to regain balance during models optimization, reducing the likelihood to overfit the majority class. With a large enough sample size, a multi-class classification approach might be considered and under/over sampling techniques could be applied for the classification of MS patients across the four MS groups, which may offer a useful tool for clinical applications. However, the aim of this study was more limited to binary classification and focused to the question of whether GM tissue degeneration, combined with the connectome representation, can discriminate MS subgroups. Thus, in order to provide comparable results with previous works, in this study, binary comparisons between MS clinical profiles were considered. Second, the conditions followed in this study for binary thresholding represent our best attempt to empirically solve the binarization problem without inducing bias in the process. The statistical analysis was in fact performed independently from the predictive classification task, in which the thresholding value was considered as an additional hyper-parameter, optimized during cross validation. Good properties were observed for thresholding values between 0.6 and 0.8. It is important to notice that, the study of GM connectome thresholding remains an open issue in the literature. In this work, we provided some empirical justifications to the thresholding rule applied for graph characterization based on the analysis performed in Section 4.1. Additionally, the statistical results remain unchanged for different nearby thresholding values ensuring robustness around the thresholding neighbours. Third, the classification results might be improved by including lesion filling during the GM segmentation, which has demonstrated to increase the accuracy of cortical thickness measurements in MS patients (Magon et al., 2014). However, this approach requires lesions to be segmented by experts, which represents a time consuming and expensive procedure. Automated lesion segmentation procedures are today available, although imprecise segmentation may hinder the improvement provided by lesion filling, thus limiting its applicability in clinical practice.

## 6 Conclusion

Although MS is mainly considered as an inflammatory and demyelinating WM disease, it also exhibits extensive GM involvement and neuro-degenerative processes. An automated pipeline was proposed in this study to characterize GM graphs, extracted from T1w MRI, using morphological features such as GM thickness. The statistical analysis revealed that significant differences were present between multiple global metrics, highlighting the importance of GM connectome graphs. The analysis was performed using two different resolution atlases, showing slightly higher classification performances with the more refined GM parcellation. The results obtained in this work are of great interest considering that only the anatomical T1w image was needed for classification of MS clinical profiles, which represents the most common MRI modality in clinical applications. To the best of our knowledge, no other studies performed classification of MS clinical profiles only considering the GM tissue degeneration measured by the morphological thickness atrophy. Notwithstanding, the use of more advanced Deep Learning (DL) methods may provide an additional improvement to the baseline results proposed in this work. In particular, advances in new deep learning architectures, such as Transformer-based models and other deep learning based architectures exploiting the attention mechanisms, have demonstrated already impressive results which are worth exploring. Besides deep learning applications, connectome data analysis represents a new interesting field for studying the architectural organization of the human brain network. We plan to extend the present analysis considering a multi-view kernel-based tensor factorization approach for the fusion of multiple morphometric features extracted from the GM tissue, such as GM curvature and area. Finally, the information obtained from the WM and GM tissue may be combined in order to enhance the classification performance of the ML model. Such an approach can be performed by embedding the WM and GM connectomes in a unified graph representation, exploiting the complementary information provided by different brain tissue types. Notwithstanding, such an approach requires the acquisition of DTI data, which is less exploited for clinical applications. Also, the study of longitudinal changes in MRI biomarkers across the four groups represents an interesting evolution of the present work that we aim to investigate.

## Data Availability

The raw data supporting the conclusions of this article will be made available by the authors upon reasonable request.
